# Coping with Environmental Eukaryotes; Identification of *Pseudomonas syringae* Genes during the Interaction with Alternative Hosts or Predators

**DOI:** 10.3390/microorganisms6020032

**Published:** 2018-04-21

**Authors:** Federico Dorati, Glyn A. Barrett, Maria Sanchez-Contreras, Tanya Arseneault, Mateo San José, David J. Studholme, Jesús Murillo, Primitivo Caballero, Nicholas R. Waterfield, Dawn L. Arnold, Liz J. Shaw, Robert W. Jackson

**Affiliations:** 1 School of Biological Sciences, University of Reading, Reading, RG6 6UR, UK; f.dorati83@gmail.com (F.D.); tanya.arseneault@canada.ca (T.A.); matsjg@gmail.com (M.S.J.); r.w.jackson@reading.ac.uk (R.W.J.); 2 Department of Biology and Biochemistry, University of Bath, Bath, BA1 9BJ, UK; mariasancon@gmail.com (M.S.-C.); n.r.waterfield@warwick.ac.uk (N.R.W.); 3 School of Biosciences, University of Exeter, Exeter, EX4 4QD, UK; d.j.studholme@exeter.ac.uk; 4 Instituto de Agrobiotecnología, Universidad Pública de Navarra, 31192 Mutilva, Spain; jesus.murillo@unavarra.es (J.M.); pcm92@unavarra.es (P.C.); 5 Warwick Medical School, University of Warwick, Warwick, CV4 7AL, UK; 6 Centre for Research in Bioscience, Faculty of Health and Applied Sciences, University of the West of England, Bristol, BS16 1QY, UK; dawn.arnold@uwe.ac.uk; 7 Agriculture and Agri-Food Canada, Saint-Jean-sur-Richelieu, Research and Development Centre, Quebec, J3B 3E6, Canada; 8 School of Archaeology, Geography and Environmental Science, University of Reading, Reading, RG6 6AX, UK; e.j.shaw@reading.ac.uk

**Keywords:** *Pseudomonas syringae*, rapid virulence annotation, RVA, pathogen, anti-predation, *Caenorhabditis elegans*, *Acanthamoeba polyphaga*, *Galleria mellonella*

## Abstract

Understanding the molecular mechanisms underpinning the ecological success of plant pathogens is critical to develop strategies for controlling diseases and protecting crops. Recent observations have shown that plant pathogenic bacteria, particularly *Pseudomonas*, exist in a range of natural environments away from their natural plant host e.g., water courses, soil, non-host plants. This exposes them to a variety of eukaryotic predators such as nematodes, insects and amoebae present in the environment. Nematodes and amoeba in particular are bacterial predators while insect herbivores may act as indirect predators, ingesting bacteria on plant tissue. We therefore postulated that bacteria are probably under selective pressure to avoid or survive predation and have therefore developed appropriate coping mechanisms. We tested the hypothesis that plant pathogenic *Pseudomonas syringae* are able to cope with predation pressure and found that three pathovars show weak, but significant resistance or toxicity. To identify the gene systems that contribute to resistance or toxicity we applied a heterologous screening technique, called Rapid Virulence Annotation (RVA), for anti-predation and toxicity mechanisms. Three cosmid libraries for *P. syringae* pv. *aesculi*, pv. *tomato* and pv. *phaseolicola*, of approximately 2000 cosmids each, were screened in the susceptible/non-toxic bacterium *Escherichia coli* against nematode, amoebae and an insect. A number of potential conserved and unique genes were identified which included genes encoding haemolysins, biofilm formation, motility and adhesion. These data provide the first multi-pathovar comparative insight to how plant pathogens cope with different predation pressures and infection of an insect gut and provide a foundation for further study into the function of selected genes and their role in ecological success.

## 1. Introduction

A major challenge in securing global food security is to reduce the impact of disease in food and resource plants (e.g., trees), and to prevent the emergence of new pathogens. Much of the seminal work in the understanding of plant resistance has relied upon a foundation of knowledge of the role and functionality of pathogen systems (e.g., type III protein secretion). However, a poorly understood branch of pathogen biology is their wider ecological success and epidemiology. How does a pathogen survive *ex planta* after an infected plant dies and deposits its tissue, with high titers of pathogen, into soil or water?

Recent work by Morris and colleagues has discovered that plant pathogens can be isolated from a wide range of environments, can be highly dispersed and are often genetically distinct [1,2]. For example, *Pseudomonas syringae* can be found from clouds to snow pack and river courses [3,4]. This highlights a major gap in our knowledge, particularly when considering functional studies—how do the pathogens survive when not in their host plant? This question can be addressed at many levels, for example, are there different plant hosts, animal hosts, or abilities to tolerate vastly different chemical environments? Thus, an understanding of the ecology of plant pathogens and discovering the mechanisms underpinning bacterial survival in the environment is critical both for increasing knowledge in bacterial evolution and to prevent or control dispersal. 

The plant-pathogenic bacterium *Pseudomonas syringae* is regarded as the most important model pathogen for use in the study of bacterial plant disease [5]. It has recently been the central focus for studying plant pathogen ecology outside of its “host” plant, having been discovered in a range of different habitats often independent from the sphere of agricultural production influence [6]. *P. syringae* can be transmitted into the atmosphere through the evapotranspiration of water from plants and reach the clouds at several kilometers in altitude [7]. From the atmosphere *P. syringae* can eventually bionucleate water to cause precipitation and fall with rain into a variety of environments [3,8]. Even though *P. syringae* can be found in the most disparate environments, it is still not clear how it survives or to what extent it undergoes evolutionary change. This latter point is particularly interesting because different stresses imposed on bacteria by changes in the environment or different biotic interactions can drive evolutionary change [9,10,11]. Moreover, the interaction with a wide range of bacteria can also open up a vast pool of genetic resources for horizontal gene transfer [12].

One area of particular interest is an understanding of how *P. syringae* can cope with predation stress. Nematodes, amoeba and insects are common inhabitants of soil, water courses and plants, so *P. syringae* will frequently encounter these predators and have likely evolved survival strategies. Nematodes and insects in particular are commonly used models for studying animal pathogens. Nematicidal activity has been reported for several environmental bacteria including *P. aeruginosa* [13]. *P. aeruginosa* can kill *Caenorhabditis elegans* in a few days (slow killing) when grown on minimal nutrient-poor medium, or in a few hours (fast killing) when grown on a nutrient-rich medium [14]. Modulating the virulence can be advantageous in vivo as the host can be used as a vector for spreading or surviving in adverse environmental conditions. More recently, a screen of several *Pseudomonas* species found a range of impacts on nematode development, killing and deterrence [15]. In that particular analysis, several *P. syringae* strains were observed to have weak or no effect on *C. elegans* egg laying or grazing. 

Free-living protozoa, such as amoebae, are widespread in the environment, especially in water and can act as environmental reservoirs of food-borne pathogens [16]. Amoebic passage of *Legionella pneumophila* is reported to enhance bacterial survival [17]. *Salmonella enterica* can, for example, survive within *Acanthamoeba polyphaga* and *A. rhysodes* [18]. Recently, the SPI-1 type III protein secretion system has been implicated in *P*. *fluorescens* F113 resistance to amoeboid grazing [19].

In insects, *P. syringae* can survive ingestion by the pea aphid *Acyrthosiphon pisum* [20]. After ingestion, *P. syringae* replicated in the gut of the aphids and were subsequently secreted in the honeydew at a considerable distance from the original ingestion point, thus demonstrating an effective dispersal mechanism for the pathogen. This interaction appears to be a common trait in other plant pathogens, with *Dickeya* and *Erwinia* also able to survive ingestion and kill aphids [21,22]. A more common insect model used as an alternate host to analyse infection is that of the waxmoth *Galleria mellonella*. While this is usually used in a non-feeding state, by direct injection of bacteria into the haemocoel, it has nonetheless proved useful for identifying virulence factors [23,24]. One study found that *P. fluorescens* CHA0 and Pf-5 could kill *G. mellonella* after injection of the bacterium into the insect larvae [25]. This was found to be due to a single locus encoding the *Photorhabdus luminescens mcf*-like toxin *P. fluorescens* insecticidal toxin (Fit).

Identifying the genetic basis for bacterial interactions with alternative hosts, including predators, requires innovative methodologies. One such technique, a heterologous screening assay called Rapid Virulence Annotation (RVA) [26] was developed to identify putative bacterial genes conferring Gain of Toxicity (GOT) to the nematode *C. elegans* (nGOT), the protozoan *A. polyphaga* (aGOT) and the larvae of the wax-moth *G. mellonella* (iGOT). RVA has already been successfully adopted to find virulence islands in the dual insect and human pathogen *Photorhabdus asymbiotica* [26].

Taking advantage of the tools available to us, we therefore sought to characterize the interaction of three different *P. syringae* strains with *C. elegans*, *A. polyphaga* and *G. mellonella*. Once we established that the there was evidence of survival to the predators and toxicity in *G. mellonella*, we then carried out an RVA screen to identify candidate *P. syringae* cosmid libraries to find genes conferring survival/toxicity. Several genes putatively involved in the survival of predation and insect toxicity were examined to gain novel insight to how *P. syringae* can be surviving interactions with predators and alternate hosts.

## 2. Materials and Methods

### 2.1. Bacterial Strains, Plasmids, Eukaryotes and Culture Conditions

Bacterial strains and other organisms used in this study are listed in Table 1. *Pseudomonas* strains (*P. syringae* pv. *tomato* (*Pto*), *P. syringae* pv. *phaseolicola* (*Pph*) and *P. syringae* pv. *aesculi* (*Pae*)) were grown at 25 °C in King’s Medium B (KMB) [27] and Luria Bertani (LB) broth [28]. *E. coli* strains were grown at 37 °C in LB media or broth. Antibiotics and supplements (Sigma-Aldrich, Gillingham, UK) were used to the following final concentrations: kanamycin 25 μg mL^−1^; tetracycline 10 μg mL^−1^; nitrofurantoin 10 μg mL^−1^. Bacterial cell titers for experiments were calculated using OD readings where a broth of OD_600_ value of 1.2 equates to 10^9^ cfu mL^−1^.

*C. elegans* were routinely propagated every week on nematode growth medium (NGM) [15] seeded with *E. coli* OP50 and incubated at room temperature. For sub-culturing a loop was used to collect nematodes from a week-old plate and transferred to a fresh NGM agar plate spread with OP50. *Acanthamoeba polyphaga* was grown in peptone yeast-extract glucose (PYG) medium [18] as a monolayer in a 50 mL Falcon tube at 23 °C. *A. polyphaga* was sub-cultured weekly by gently tapping flasks to detach cells before diluting 1:10 in fresh PYG medium. Stationary phase (3–5 days) cultures of *A. polyphaga* were used throughout this study by the following approach. *A. polyphaga* was harvested by gently tapping flasks, followed by centrifugation (3000 rpm, 5 min) and washed twice in Page’s amoeba saline (PAS) (2.5 mM NaCl, 1 mM KH_2_PO_4_, 0.5 mM Na_2_HPO_4_, 40 mM CaCl_2_, and 20 mM MgSO_4_) solution, before numbers were adjusted to 2 × 10^5^ cells mL^−1^ using a haemocytometer for counting samples. *G. mellonella* were obtained from Livefood UK at L4 stage. The moth larvae were kept in the fridge at 4 °C for no longer than two weeks before their use, and once used in the experimental tests, kept at room temperature.

### 2.2. Survival Assays against P. syringae Strains

#### 2.2.1. *Caenorhabditis elegans* Killing Assay

Approximately 100 μL of an overnight culture of a *P. syringae* strain was spread onto NGM in 5.5 cm Petri plates and incubated at 27 °C for 24 h. After an additional 24 h at room temperature (23–25 °C) each plate was seeded with ten L4-stage hermaphrodite *C. elegans* nematodes. Plates were incubated at 25 °C and examined for *C. elegans* viability every 24 h for 3 days. After incubation each *C. elegans* adult was assessed for response to touch (an indicator of life status). Non-movers following touching were scored as dead.

#### 2.2.2. *Acanthamoeba polyphaga* Growth Assay

A population of 5 × 10^4^ cells of *A. polyphaga* amoebae cells, previously grown for 4 days in PYG medium, were co-incubated for 0, 12 and 24 h with 2 × 10^2^ cfu mL^−1^ *P. syringae* in phosphate buffered saline (PBS) [28]. *E. coli* OP50 was used as positive control. At the end of the co-incubation amoebae were sedimented by gentle centrifugation at 200 rpm for 10 min, rinsed three times in sterile water, re-eluted and serially diluted in PBS. Live amoebae were counted using a haemocytometer and original populations calculated using the dilution factor giving the most statistically accurate population count.

#### 2.2.3. *Galleria mellonella* Killing Assay

A U-100 insulin syringe equipped with a 29-gauge needle (BD) was used to inject 10 μL aliquots of a 1 × 10^6^ cfu mL^−1^ bacterial inoculum in PBS or 10 mM MgCl_2_ into the haemocoel of each *G. mellonella* larvae via the last left proleg. Before and after injection, the area was cleaned using an alcohol swab. After injection, *G. mellonella* larvae were incubated in plastic containers and kept at room temperature in the dark. The number of dead larvae were scored daily for 72 h. Larvae were considered dead when they displayed no movement in response to a stab to the head. The crude cell-free supernatant (derived by centrifuging and filtering (0.2 μM)) from an overnight (O/N) broth culture of the bacterial strains was also used to assess toxicity.

### 2.3. Survival Assay of P. syringae against Caenorhabditis elegans and Acanthamoeba polyphaga In Vitro

Overnight broths (10 μL) of *Pae*, *Pph* or *Pto* were spotted onto individual 1 mL NGM agar volumes in 25 well plates (compartmentalized Sterilin^TM^ 100 mm square Petri dishes, Thermo Scientific, Basingstoke, UK) and grown overnight. The following day *C. elegans* nematodes were washed from a 4-day old NGM plate using 1 mL PBS and a sterile glass spreader. Nematodes per unit volume were enumerated using light microscopy and approximately 30 adults were added into the top right corner of each well in the plate. Bacteria and nematodes were recovered from the plate after 0, 7 and 14 days using 1 mL 10 mM MgCl_2_, homogenized using sterile micropestles (Eppendorf, Hamburg, Germany). Samples were serially diluted in sterile water, plated on KMB supplemented with nitrofurantoin and bacterial colonies enumerated. Samples were done in triplicate for each time point. *E. coli* OP50 was used as a control strain. The same experiment was performed using *A. polyphaga* as predator. An aliquot of 10 μL overnight broths of *Pae*, *Pph* or *Pto* were inoculated on PYG medium poured in 25 well plates (each well containing approximately 1 mL of medium) and grown overnight. The following day 10 μL of a 7-day old culture of *A. polyphaga* (approximately 3 × 10^5^ cells) were spotted in the centre of each well. Samples were done in triplicate for each time point. The bacteria and the *A. polyphaga* were recovered from the plate after 0 and 7 days using 1 mL 10 mM MgCl_2_, serially diluted in sterile water, plated on KMB and the bacteria counted. *E. coli* OP50 was used as a control strain.

### 2.4. Molecular Biology Techniques including Cosmid Library Creation and Sequencing

Cosmid DNA was isolated from a pure bacterial culture using either a mini or midi prep kit (QIAGEN, Manchester, UK) and restriction enzymatic digests performed as per manufacturers protocols (NEB). Total DNA was extracted with a Puregene kit for Gram-negative bacteria (QIAGEN). A *Pae* P6617 cosmid library (1800 clones) was constructed using cosmid vector pIJ3200 as previously described [33] whereas two pLAFR3-based cosmid libraries (2000 clones each) of *Pph* 1448A and *Pto* DC3000 were already in existence. Each cosmid clone had an insert of between 30–40 kb for the 1448A and DC3000 libraries and 10–40 kb for the *Pae* library, thus providing between 7.5 to 13-fold genome coverage. Primers used in construction are listed in Supplementary Table S1. The end-sequencing for the cosmid was performed by Geneservice (Source BioScience, Oxford, UK) using primer pair pLAFR3Forint and pLAFR3IntRev for the library of *Pph* and *Pto* which was constructed in pLAFR3 and primer pair T3 and T7 for the *Pae* library constructed in pIJ3200. Standard PCR reactions were performed using GoGreen Master mix (Promega, Madison, WI, USA) whereas BIO-X-ACT Short polymerase (Bioline, London, UK) was used for high fidelity amplifications; oligonucleotide primer pairs used per respective assay are listed in Supplementary Table S1. PCR fragments were purified from agarose gels by QIAquick columns (QIAGEN).

### 2.5. RVA Analysis of Gain of Toxicity (GOT) Assays

We adapted the Gain of Toxicity (GOT) method of Waterfield et al. [26] to carry out screens of *P. syringae* cosmid libraries in the presence of nematode or amoeba predation, or by injection to *G. mellonella* larvae. This technique occurs by screening fragments of a host genome (in this instance, cosmids containing genomic sections) within a susceptible host bacterium in the presence of the *C*. *elegans* or *A. polyphaga* predators, or within the haemocoel of the *G. mellonella* larvae. The methodology for each screening system is described below. Cosmids conferring Medium-Resistant survival/toxicity were chosen for end-sequencing. The end sequences were aligned to the genome sequences of the relevant strains using NCBI BLAST, and viewed using Artemis and Artemis Comparison Tool, allowing the cosmid gene content to be manually assessed. To simplify the downstream analysis, a scoring system was devised to rank cosmids where overlapping cosmids from the same GOT or cosmids that were selected from multiple GOT were priority targeted. A value of “1” was given to a gene every time it was represented in any of the GOT screens. Scoring higher than “3” (Supplementary Table S2) were considered for further “in silico” analysis to investigate the gene functions in detail.

#### 2.5.1. *Caenorhabditis elegans (nGOT)*

*C. elegans* was grown for 4 days at 18 °C in NGM seeded with *E. coli* OP50. The *P. syringae* cosmid libraries were grown in 96 wells plates on LB supplemented with tetracycline overnight and 10 μL of each cosmid clone was placed in the middle of each well in a 25 well plate containing NGM supplemented with tetracycline. The plates were incubated at 37 °C O/N. *C. elegans* nematodes were washed from plate cultures, as above, using PBS and washed three times in PBS. The density of the nematodes was adjusted to 40 nematodes per 10 μL and spotted in the corner of each well of the 25 wells per plate containing the library clones. The plates were incubated at room temperature and assessed over 11 days. The resistance of the cosmid colony in each plate was scored based on disappearance of the bacterial colony: disappearance after 1 day (Very Low resistance), 3 days (Low), 5 days (Medium), 7 days (High), 9 days (Very High); still present at 11 days (Resistant). 

#### 2.5.2. *Acanthamoeba polyphaga (aGOT)*

*A. polyphaga* was grown at 25 °C for 7 days in PYG media and then diluted in fresh PYG media to reach a concentration of 2 × 10^5^ cells mL^−1^. Forty eight colony arrays of each *P. syringae* cosmid library were replica plated onto 9 cm Petri dishes containing PYG agar [26] supplemented with tetracycline and grown at 37 °C overnight. The following day 10 μL volumes of *A. polyphaga* were spotted to each centre location within the array (i.e., 35 *A. polyphaga* spots within the 48 arrayed bacterial strains/clones) and incubated for up to 12 days at room temperature. The assessment started at 3 days, whereby plates were scored each day. The resistance of the cosmid colony in each plate was scored based on disappearance of the bacterial colony: disappearance after 3 days (Very Low resistance), 5 days (Low), 7 days (Medium), 9 days (High); 11 days (Very High); still present after 11 days (Resistant).

#### 2.5.3. *Galleria mellonella (iGOT)*

The *P. syringae* cosmid libraries were grown in 96 well plates on LB supplemented with tetracycline at 37 °C overnight and 5 μL of each cosmid was injected by dipping a 25-gauge needle into a broth and stabbing the needle into the open blood system (haemocoel) of *G. mellonella* larvae in triplicate. The larvae were then kept in 25 well plates at room temperature and scored for 4 days. Colonies that caused mortality or morbidity or altered the health (melanization, and sluggish movement) of *G. mellonella* were retested. The toxicity of the cosmid colonies was scored based on time to death: larval death after 12 h (scored 1), 1 day (scored 2), 2 days (scored 3), 3 days (scored 4); 4 days (scored 5); still alive (scored 6). The approximate number of bacteria injected was pre-assessed by dipping the needle in an overnight broth, flushing the needle in sterile water, serially diluting in sterile water and plating onto LB agar supplemented with tetracycline.

### 2.6. Gene Knock-Out Strategy to Create Mutants

Knock-out mutants of *P. syringae* were constructed as follows: approximately 400 bp of DNA internal to each gene was amplified using a proofreading PCR enzyme and ligated into the vector pCR 2.1_TOPO. Each ligation was electroporated into electrocompetent *E. coli* TOP10 cells (Invitrogen, Carlsbad, CA, USA). Recombinant colonies were selected by antibiotic resistance, and the knock-out mutation was confirmed by PCR analysis using a combination of vector primers and gene specific primers.

### 2.7. Biofilm and Swarming Characterisation of Mutants

The biofilm formation assay was performed in 96-well polystyrene microtiter plates. The bacterial strains to be tested were grown overnight at 25 °C or 37 °C in LB broth and diluted 1:100 in fresh medium (to achieve 10^7^ cfu mL^−1^) into the 96-well plates the following morning. The plates were subsequently incubated at 27 °C for 24 h without shaking. The amount of biofilm formation was determined by pouring out the contents of the wells and adding 200 μL of 1% crystal violet to each well for 1 min before rinsing with water and eluting stain with 100% EtOh for 5 min. The level of stain retained by cells was quantified by measuring the absorbance at 595 nm. The swarming assay was performed using LB medium supplemented with 0.5% Bacto-Agar. The bacterial strains to be tested were transferred from an overnight culture plate using a sterile toothpick and cells stabbed into the centre of the soft agar; plates were incubated at 27 °C. The assays were performed independently at least three times and motility was scored as the distance in cm that the bacteria moved from the inoculum point to the edge of the growing area. Motility was assessed and recorded every 24 h for 7 days.

### 2.8. Statistical Analyses

Statistical analyses were performed using 2- (time and bacterial treatment) or single factor ANOVAs (bacterial treatment) using SAS statistical software (v 9.4) (Cary, MT, USA). Most analyses were followed by Tukey-Kramer adjusted multiple comparisons of the data, except for the characterization of the mutants (Figure 4), for which planned contrasts were made between each mutant and the *Pto* and *E. coli* controls thereby rendering comparisons as inconsequential between individual mutants.

## 3. Results

### 3.1. Pseudomonas syringae Strains Affect the Survival of Caenorhabditis elegans, Acanthamoeba polyphaga and Galleria mellonella

We opted to study three *P. syringae* pathovars, *Pto* DC3000 a tomato pathogen, *Pae* P6617 a tree pathogen and *Pph* 1448A, a bean pathogen. Each strain was originally isolated from very different environments and we postulate that as such they may have evolutionary adaptations for those niches. Furthermore, genomic analysis of these three pathovars *Pto* DC3000 [29], *Pae* P6617 [30] and *Pph* 1448A [31] has revealed they are also phylogenetically distinct. Our first analysis was to determine the outcome of the wild-type bacterial interaction with each predator taxa. Each assay differed slightly in the measured outcomes due to the nature of the organism being used; nematodes were tested by feeding and touch response, amoeba by grazing and increase in numbers, *Galleria* larvae by injection and touch response.

**Figure 1:**
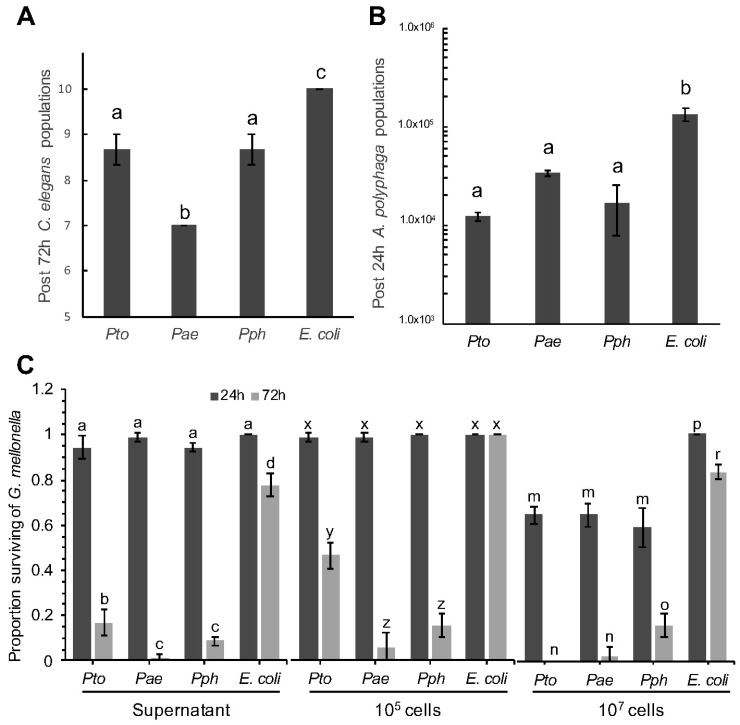
Temporal effect of *Pseudomonas syringae* strains on population sizes of *Caenorhabditis elegans* and *Acanthamoeba polyphaga*, and mortality of *Galleria mellonella*. (**A**) With initial populations of 10 L4 stage *C. elegans*, data shows number of *C. elegans* alive after 72 h, when challenged with *Pto*, *Pae*, *Pph* and *E. coli* control. ANOVA detected statistically significant differences (*p* = 0.0002, df = 8); (**B**) With initial populations of 1.2 × 10^4^ *A. polyphaga* cells, data shows numbers of *A. polyphaga* cells after 24 h when co-cultured in presence of *Pto*, *Pae*, *Pph* and *E. coli* control. ANOVA detected statistically significant differences (*p* = 0.0008, df = 8); (**C**) Quantitative analysis of *G. mellonella* mortality by *P. syringae*, survival rate represents the relative number. Treatment type (supernatant, 10^5^ and 10^7^ cells), was analyzed separately by 2-factor ANOVA. An interaction between time and *P. syringae* treatments resulted in multiple comparisons for all types of treatment (*p* < 0.0001, df = 16). For all panels, bars represent the mean of 3 replicates, error bars represent standard error of the mean. Comparison of means by Tukey-Kramer HSD are shown as letters above bars, where different letters indicate statistically significant differences (*p* < 0.05).

Ten L4 stage *C. elegans* adults were incubated with a lawn of *Pae*, *Pto*, *Pph* or *E. coli* OP50 on an NGM agar plate for 72 h. All three *P. syringae* strains exhibited weak, yet statistically better, killing of *C. elegans* compared to the *E. coli* OP50 control. *Pae* was most effective, killing 3/10 (30%) of the nematodes compared to 1.3/10 (13%) for both *Pto* and *Pph* (Figure 1A). In bacteria and amoeboid co-cultures, and starting from known inoculation loads, *A. polyphaga* populations after 24 h, whilst higher in each respective case, were significantly lower when in the presence of the *P. syringae* strains compared with *E. coli* OP50 (Figure 1B). 

The final test was to assess the effect of bacteria on insect larvae after injection. Both bacterial cells and cell-free supernatant from an overnight bacterial culture were injected into the haemocoel of *G. mellonella* and larval mortality scored after 24 and 72 h (Figure 1C). All the *P. syringae* strains triggered a strong immune response after 8 h as evidenced by melanisation of the larvae; followed by death after 72 h. The extent of the response was observed to be dose dependent, with the higher titre of cells resulting in increased mortality. All three *Pseudomonas* strains led to significantly higher numbers of dead larvae compared to the *E. coli* OP50 negative control strain. The supernatant from an overnight culture of the same strains was also used to assess if any toxic compound(s) had been released by the bacterial cells into the extracellular milieu. In these experiments a similar pattern of toxicity was observed 72 h post-injection i.e., supernatants from all *P. syringae* strains were statistically more toxic than *E. coli*. This is consistent with the hypothesis that at least some component(s) of the *P. syringae* pathological effects may be the result of secreted virulence factor(s).

### 3.2. Pseudomonas syringae Survival against Caenorhabditis elegans and Acanthamoeba polyphaga

**Figure 2:**
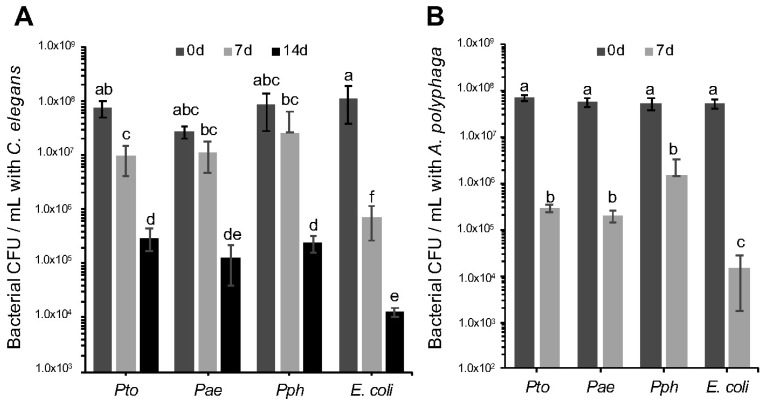
Survival of *Pseudomonas syringae* strains upon predation by *Caenorhabditis elegans* and *Acanthamoeba polyphaga*. (**A**) *Pto*, *Pae* and *Pph* were assessed for survival upon *C. elegans* predation. Bacterial populations were assessed after 0, 7 and 14 days of co-incubation with 10 L4 stage *C. elegans*. A 2-factor ANOVA detected statistically significant differences and showed an interaction between the time and treatment factors (*p* = 0.0007 df = 24); (**B**) *Pto*, *Pae* and *Pph* were assessed for survival when challenged with *A. polyphaga*. Bacterial population were assessed after 0 and 7 days of co-incubation with 3 × 10^5^ *A. polyphaga* cells. ANOVA detected statistically significant differences (*p* = 0.0002 df = 16). For all panels, bars represent the mean of 3 replicates and error bars indicate the standard error of the mean. Multiple comparison of means by Tukey-Kramer HSD are shown as letters, where different letters indicate statistically significant differences (*p* < 0.05).

It is a reasonable hypothesis to propose that the negative impact of the *P. syringae* strains on *C. elegans* relates to strategies that have evolved to survive predation in the environment. Limiting toxicity to the host but at the same time surviving predation can be an important step for bacterial dispersion in the environment and colonization of new hosts [34]. Therefore, we decided to evaluate if the bacterial cells themselves were able to directly resist predation. To test strain survival following predatory challenge with *C. elegans*, 10 μL of 1 × 10^8^ cfu mL^−1^ *Pae*, *Pto*, *Pph* and *E. coli* OP50 were co-incubated on an NGM agar plate together with 10 L4 stage *C. elegans* adults. Sets of plates were incubated at 23 °C for 7 and 14 days after which time the bacteria were recovered and the survivors enumerated. Significant differences in survival of the *P. syringae* strains compared to *E. coli* OP50 were observed after 7 and 14 days (Figure 2A). These results clearly indicate that nematode predation does reduce all the bacterial populations tested, but also that all three *P. syringae* strains tested were capable of resisting *C. elegans* predation to a greater degree than *E. coli* OP50. Furthermore, in experiments designed to assess the impact of *A. polyphaga* grazing, we again observed that while the amoebae were able to reduce numbers of all bacterial strains tested, the *E. coli* OP50 control exhibited a larger reduction in population size compared to the *P. syringae* strains (Figure 2B). Taken together, these results are consistent with the hypothesis that *P. syringae* has evolved anti-predation mechanisms for nematodes and amoeba, and also secretes one or more virulence factors toxic to an insect.

### 3.3. RVA Analysis of Pseudomonas syringae pv. tomato, Aesculi and Phaseolicola Cosmid Libraries Determine Gain of Toxicity (GOT)

**Figure 3:**
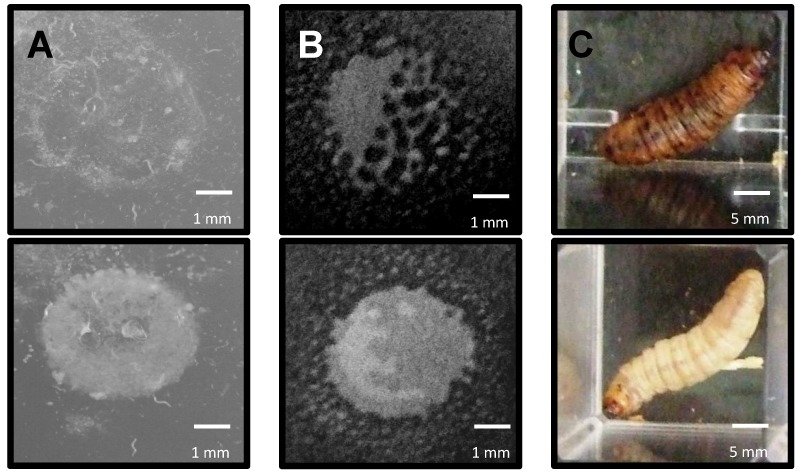
Examples of observed predation by *Caenorhabditis elegans*, *Acanthamoeba polyphaga* and gain of toxicity (GOT) against *Galleria mellonella* of bacterial strains during RVA assays. (**A**) nGOT assay. Colonies carrying *P. syringae* genes (as cosmid clones) not involved in survival were eaten by *C. elegans* within 72 h (top) but those with genes involved in resistance were able to resist predation for longer (bottom); (**B**) aGOT assay. Colonies carrying genes not involved in survival were gradually eliminated by *A. polyphaga* within 7 days (top), but those which carried genes involved in resistance were able to resist predation for longer (bottom); (**C**) iGOT assay. Colonies carrying genes conferring an increase in toxicity were capable of killing *G. mellonella* within 48 h (top) while others were unable to kill the larvae (bottom). Scale bars in white.

Earlier experiments indicated that all *P. syringae* strains were phenotypically better at either surviving predation by nematodes or amoeba, or killing *Galleria*, than *E. coli* OP50. To identify the genetic basis of these differences, the cosmid libraries of the three *P. syringae* strains were screened in each GOT assay, assessing individual clones for *E. coli* GOT. For both *Pto* and *Pph*, 2000 cosmid clones were screened while 1800 were screened for *Pae*. The GOT assays used were identified as nGOT (using the nematode *C. elegans*), aGOT (using the amoebae *A. polyphaga*) and iGOT (using the insect *G. mellonella*). The ability to survive predation by either *C. elegans* or *A. polyphaga* is shown in Figure 3A,B respectively, while the *G. mellonella* killing assay is shown in Figure 3C.

Based on the GOT assays, the *Pto* library yielded the highest number of predation resistant clones for each predator. Out of the 2000 cosmid clones tested in each GOT screening, 201 *Pto* clones were scored as positive for increasing *E. coli* resistance to predation. We obtained 99 positive clones from the *Pae* library and 74 from the *Pph* library (Table 2). These differences point to the already established genetic variation between strains, or alternatively, that there were distinct levels of enrichment of particular genomic regions during the library construction process. If the results are analyzed in respect to the specific GOT assays we see that the *Pph* and *Pae* cosmid libraries showed a higher number of cosmids conferring resistance against *A. polyphaga* followed by comparable numbers showing resistance against *C. elegans* and toxicity against *G. mellonella*. Conversely, the number of clones obtained from the *Pto* library were relatively higher against *G. mellonella*, but lower against *C. elegans* and *A. polyphaga*. Again, it is not clear if this reflects different genomic content of the strains or strain specific cryptic bias in cosmid construction.

### 3.4. Sequencing and Bioinformatic Analysis of RVA’s Positive Cosmid Clones

The cosmid clones scoring a positive phenotype of medium resistance/toxicity (or greater) from the RVA screening were selected for insertion-end sequencing. Sanger sequencing, using the forward and the reverse primers on the cosmid vector was used to determine the first 800–1000 bp of each cosmid insert. Overall 65 cosmid clones were successfully sequenced from the iGOT assay, 24 from the aGOT assay and 34 from the nGOT assay. Prioritization of analysis was given to those cosmid clones that scored as a 3 (see Section 2), whereby common genes were emerging from the GOT screens. This indicated that a number of individual *P. syringae* genes or gene systems were contributing to *E. coli* survival or toxicity. Another interesting observation was that several potential virulence genes were present in more than one *P. syringae* strain which may indicate that their function is conserved among the different strains. Supplementary Table S2 summarizes the best hits found from the RVA using *Pto* as a reference strain and compared for presence/absence in *Pph* and *Pae* RVA’s screening.

### 3.5. Validation and Characterisation of Genes Identified by RVA

Although the RVA analysis allows for the rapid screening of genotype/phenotype relationships, the trade-off is that each cosmid encodes for between 15–40 genes, any one or cluster of which could be conferring the GOT phenotype to the *E. coli* host strain. To rationalize a set of candidates for validation, we focused on genes that were positively scored in more than one GOT assay and for more than one strain; these were *pslA*, *fleQ*, *pilJ*, *cheW*, PSPTO_0373 (*rhsD)*, and *hlyD* (Table 3). Since we were using RVA as a tool to inform us about putative genes contributing to *P. syringae* ecological success, we focused on making insertional gene knock-outs in *Pto* strain DC3000 since this strain is particularly amenable to genetic manipulation. Insertional mutants for all genes were accomplished except for the *cheW* and *hlyD* genes. As an alternative we therefore created, *cheA* and *hlyIII* mutants to act as surrogate functional comparators. Gene complementation was not done since mutations in gene clusters (potentially causing polar effects) were deemed acceptable means of knocking out gene function as were knock-outs of orphan genes.

### 3.6. Survival and Toxicity Characterisation of Mutants against C. elegans, A. polyphaga and G. mellonella

**Figure 4:**
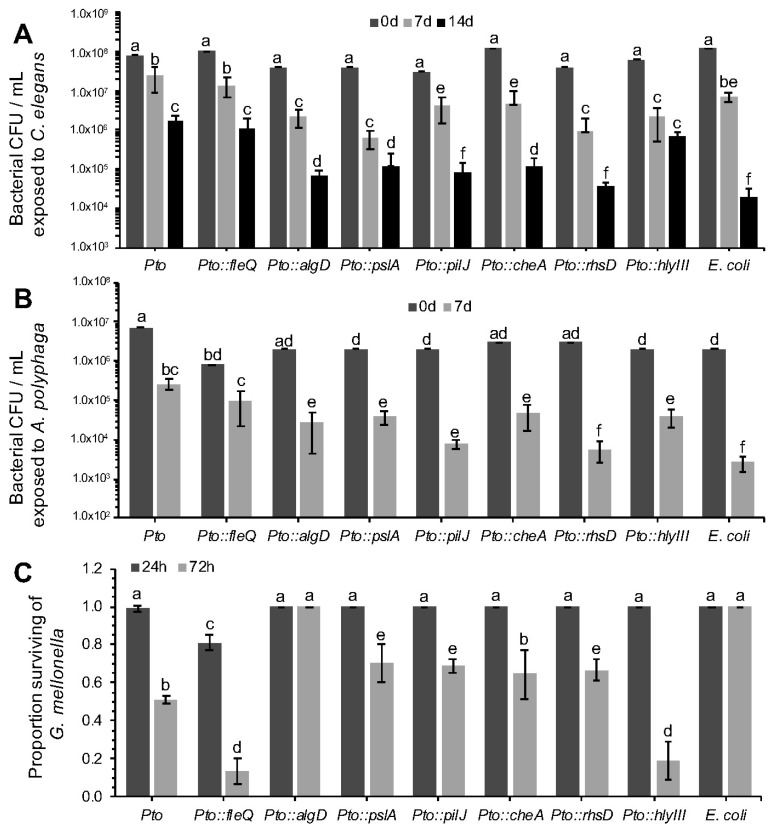
Characterization of *Pto* mutant survival after predation by *Caenorhabditis elegans* and *Acanthamoeba polyphaga* and killing of *Galleria mellonella*. See Table 3 for mutant descriptions. (**A**) Strains were assessed for survival against *C. elegans* predation. Bacterial populations were assessed after 0, 7 and 14 days of co-incubation with 10 L4 stage *C. elegans*. 2-factor ANOVAs detected statistically significant differences (*p* < 0.0001, df = 18); (**B**) Strains were assessed for survival when challenged with *A. polyphaga*. Bacterial populations were assessed after 0 and 7 days of co-incubation with 3 × 10^5^ *A. polyphaga* cells. 2-factor ANOVAs detected statistically significant differences (*p* < 0.001 df = 12); (**C**) Quantitative analysis of *G. mellonella* killing by *Pto* mutants (10^5^ cells). 2-factor ANOVAs detected statistically significant differences (*p* < 0.0001 df = 12). For all panels, bars represent the mean of 3 replicates and error bars indicate the standard deviation. Statistical differences were determined by contrasting each individual mutant to the *Pto* and *E. coli* controls, therefore different mutants cannot be compared between them but only to the controls themselves. Differences are shown as letters (*p* < 0.05).

The survival of all the mutants described in Table 3 was assessed against *C. elegans* (Figure 4A) and *A. polyphaga* (Figure 4B) using the same approach as described in Section 3.2. Their toxicity against *G. mellonella* was also assessed as described in Section 3.1 (Figure 4C). Statistical analyses using planned contrasts between each mutant and wild-type *Pto* and *E. coli* were performed in order to determine significant differences. Note, we do not believe there is any reasonable logic or statistical sense in comparing individual mutants.

In the predation assay with *C. elegans* and *A. polyphaga*, *Pto::pslA* exhibited a significant reduction in survival compared to the wild type. After seven days there was 1.5 to 2-log of difference between *Pto* and *Pto::pslA.* When tested in the *G. mellonella* killing assay statistically significant differences in mortality of the larvae were recorded between the *Pto* and *Pto::pslA*. After 72 h the *Pto::pslA* mutant was 50% less virulent than *Pto*.

The *Pto::fleQ* exhibited no difference in *C. elegans* or *A. polyphaga* grazing resistance phenotype compared to *Pto* wildtype, despite the *flg* genes being identified from the nGOT screen. However, when *Pto::fleQ* was tested in the toxicity assay against *G. mellonella* (Figure 4C), the results obtained were surprisingly different from expectations because the mutant was hypervirulent compared to the wild type. Significant differences were already noticeable after 24 h from the inoculation time as 30% of the larvae had already being killed by the mutant compared to a 1% death rate in the *Pto* wild type. After 72 h, *Pto::fleQ* had killed 87% of the larvae compared with 49% killed by *Pto*, while no death was recorded in the *E. coli* OP50 control. FleQ directly influences the expression of several genes including the alginate gene *algD* [35]. The role of alginate in plant infection is well documented [36,37], but there is no evidence of *P. syringae* using it as a virulence mechanism against invertebrates; although previous work has described alginate importance in virulence of *P. aeruginosa* [35,38]. The increased virulence of *Pto::fleQ* may be due to a hyperproduction of alginate as the down-regulation of *algD* is suppressed. To test this hypothesis, a *Pto*::*algD* mutant, unable to produce alginate, was assessed for toxicity against *G. mellonella*. *Pto::algD* completely failed to kill the larvae over a period of 72 h (Figure 4C). The data presented in this experiment appear to indicate that alginate is a product that triggers the immune system of *G. mellonella* leading to insect death. The poly-negative charge on the alginate [39] is likely to act to “mop up” innate immune cationic antimicrobial peptides [40].

*E. coli* containing cosmids carrying either the *che* (chemotaxis) or the *pil* (pilus formation) clusters had increased resistance to predation when compared to the *E. coli* wild type in the nGOT and aGOT assays. Two genes, respectively controlling pilus formation (*pilJ*) and chemotactic response (*cheA*) in *Pto* were mutated. Strain *Pto*::*pilJ* and *Pto*::*cheA* both exhibited a significant reduction in survivability compared to *Pto* during challenge with *C. elegans* and *A. polyphaga* (Figure 4).

Gene PSPTO_0373, encoding an Rhs (Rearrangement Hotspot) factor [41], was found on a cosmid that conferred increased survivability to *E. coli* when challenged by *C. elegans* and *A. polyphaga*. Rhs genes are also present in *P. aeruginosa* and other *Pseudomonas* species [42]. The function of many *rhs* elements is still unclear but some evidence suggests that in *P. savastanoi* they can facilitate bacteriocin production [43]. In *P. aeruginosa* several *rhs* elements have been shown to enhance colonisation ability of the pathogen in patients with cystic fibrosis [44]. More recently RhsT has been shown to activate the inflammasome following translocation of the protein into phagocytic cells and leading ultimately to the death of these cells [45]. When predated in vitro by *C. elegans*, *Pto::rhsD* was significantly compromised in survivability (Figure 4A). The total population decreased by more than 1 log fold compared to *Pto* in 72 h. Functional impairment in *rhsD* was even more apparent in *A. polyphaga* competition trials where, after 7 days of incubation, survivability rate of the mutant was comparable to *E. coli* OP50 and exhibited a significant decrease in cell numbers compared to the wild type *Pto* strain (Figure 4B).

The *hlyD* gene, identified in the *Pto* screen, was found to confer higher survivability to *E. coli* in the aGOT and nGOT assays. The putative product of this gene, HlyD, is related to the type I secretion membrane fusion protein, an ABC transporter. Haemolysins are a general class of toxins secreted by a range of pathogenic bacteria to lyse host cells [46]. Despite several attempts, the creation of an *hlyD* mutant was unsuccessful. However, a related haemolysin, *hlyIII* (PSPTO_5077), was identified and a *Pto::hlyIII* mutant was created and tested in a competitive assay against all three invertebrates. Survivability of *Pto::hlyIII*, compared to *Pto*, was reduced in *C. elegans* after 3 days, but not 7 days (Figure 4). Hypervirulence, similar to that seen in *Pto::fleQ,* was observed in *Pto::hlyIII* with a rapid initial die-off and a significantly higher *G. mellonella* death rate after 72 h.

### 3.7. Further Characterisation of Mutant pslA

**Figure 5:**
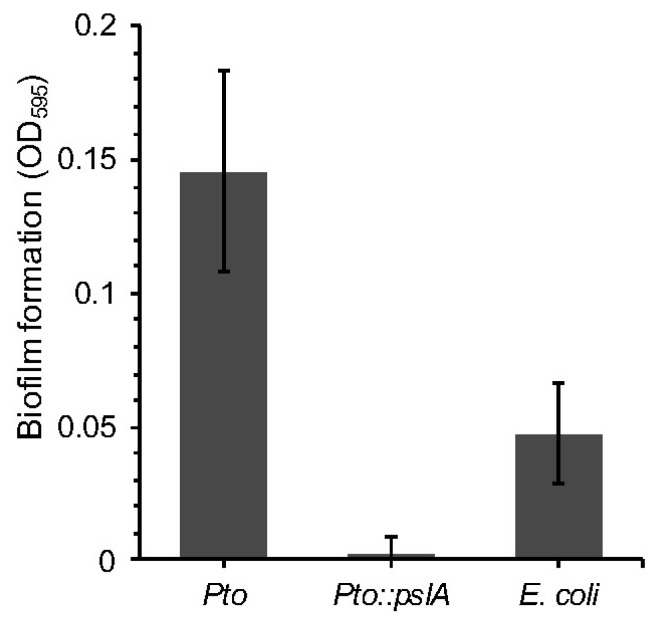
A *Pseudomonas syringae* pv. *tomato* strain DC3000 *pslA* mutant. *Pto::pslA* was assessed for the ability to form biofilms using crystal violet staining and measure of absorbance. ANOVA detected statistically significant differences (*p* = 0.0009 df = 9).

The previous experiments enabled the identification of genes potentially involved in conferring insect killing or survival to grazing. There are a raft of further tests that can now be done to understand the likely function of these gene systems. One approach would be to carry out further phenotypic analysis. For example, the RVA screening revealed that the *E. coli* cosmid clone containing the *psl* operon from *Pto* was more resistant in the nGOT assay and more toxic in the iGOT assay. The *psl* gene cluster was first identified in *P. aeruginosa* and consists of 15 genes involved in EPS (exopolysaccharide) biosynthesis and biofilm formation [47]. In *P. aeruginosa pslA* is the first gene of the operon, and knockout mutants display decreased adhesion ability and reduced biofilm formation on different surfaces [47]. *Pto* possesses a similar organization of the *psl* cluster and shares a high identity with that of *P. aeruginosa*. We can therefore hypothesize that the *pslA* cluster is important for *Pto* biofilm formation, which may be important for the nematode and insect interaction. To assess the contribution of *pslA* to *Pto* biofilm forming ability, early-stage biofilm formation was assessed by measuring adhesion of *Pto::pslA* to polypropylene. While the wildtype showed a certain level of adhesion, ability of *Pto::pslA* strain was compromised and was comparable to a sterile growth medium (LB) control, indicating, at least in this experimental context, that *psl* is crucial for biofilm production in *Pto* (Figure 5).

## 4. Discussion

The widespread occurrence of the plant pathogenic bacterium *P. syringae* in different habitats suggests the pathogen has a remarkable capacity to survive a breadth of environmental challenges. We hypothesized that one ability would be to withstand ingestion by predators and the insect haemocoel as a model for alternate hosts. We therefore aimed to determine the fate of different *P. syringae* strains, isolated from different geographical regions and host plants, after challenge with grazing predators and an insect larva. We first adapted a screening system used with an insect pathogen, *Photorhadbus asymbiotica*, to test the outcomes of challenge with nematodes, amoeba and insect larvae [26]. This involved monitoring and measuring the populations of predatory nematodes and amoeba, and also their bacterial prey numbers, after grazing on three *P. syringae* strains and for comparison a non-pathogenic *E. coli* strain, which is an innate food source for both predators. 

Nematode populations were smaller after 72 h of grazing on *P. syringae* compared to *E. coli*, but nematode growth was especially poor after challenge with the horse chestnut pathogen, *Pae*. Mean length of nematodes brought up on respective diets of the three *P. syringae* strains were shorter than those brought up on *E. coli* with *Pae*-fed nematodes being, statistically, the shortest overall (data not shown). This adds weight to the idea that the three trialed *P. syringae* isolates each had a detrimental effect on the physiology of *C. elegans* by affecting life history traits ultimately impairing development. A similar, recent study on nematodes grown on lawns of *P. fluorescens* NZ17 found that the nematodes initially investigated but then actively avoided the bacteria on the plate which led to impairment in nematode growth and indeed death of these after a few hours if conducted on certain laboratory media [15].

The differential effect of bacterial species as a food source on *C. elegans* growth and health, whether this be positive or negative, is already well characterized; and this generally being dependent on the genetic makeup of the respective bacterial species [48]. Bacteria are able to provide essential macro and micronutrients and, via excretion, a range of other peptides to support growth of *C. elegans*. For example, the quorum-sensing pentapeptide competence and sporulation factor (CSF) and nitric oxide (NO), both secreted by *Bacillus subtilis* were found to extend longevity of *C. elegans* by downregulating an insulin-like signaling pathway [49]. On the other hand, bacterial species can be highly pathogenic to *C. elegans* as demonstrated by the secretion of a number of toxins by a *Burkholderia cepacia* complex [32] which resulted in high nematode mortality. Interestingly, the authors found that nematodes pre-raised on toxic *B. cenocepacia* cells were more inclined to continue consuming these even when a co-culture of these toxic cells mixed with a non-toxic *E. coli* strain was presented implying possible manipulation of nematode feeding behavior by bacteria. This suggests that the reduced size of the nematodes in the present study is not down to an avoidance of “unsavoury” *P. syringae* cells and thus a reduction in calorie intake but indeed a physiological effect due to ingestion of these cells. It must be noted that other authors have found *C. elegans* to actively avoid toxic bacterial cells but only in preference to other food sources [50,51].

The *P. syringae* and *E. coli* populations all fell over time, indicating the nematodes were reducing the bacterial population. However, the *P. syringae* bacterial numbers were significantly higher than the *E. coli* strain, thus showing a correspondence with the nematode abundance drop. Notably, the *Pae* population was not significantly larger than the other pathovars, suggesting the differential in nematode numbers with *Pae* was more likely due to a more potent toxic effect. The bacterial–amoebal challenge reflected similarities to that seen with the nematode analysis—that amoebal populations were less effectively supported by the *P. syringae* strains compared with *E. coli*, and that the *P. syringae* populations were reduced, but to a lesser extent compared to *E. coli*. This may again point to the production and secretion of toxins, in bacterial strains encoding greater virulence mechanisms, into the extracellular milieu. Increased persistence of bacteria in soil both in terms of competition against other endogenous bacterial strains [52] and as an avoidance mechanism to protozoan grazing [53] has already been attributed to the production of potentially toxic compounds. *Pseudomonas fluorescens* CHA0, for example, produces the protease AprA which provoked significant encystation of the amoeba *Vahlkampfia* sp. upon comparison to its isogenic mutant [54].

The third test analyzed the outcome of bacterial injection to the haemocoel of *G*. *mellonella*. Although this is a somewhat artificial screening system (as the insect would not be expected to ingest these bacteria in its natural honeybee parasitism habitat), it does provide a useful model for examining bacterial survival of the insect immune system. A dose-dependent effect was observed for *P. syringae*, with all three strains rapidly killing the larvae, whereas the *E. coli* had little or no toxic effect. Interestingly, the injection of spent bacterial broth (supernatant after removal of cells) resulted in larval death, suggesting that at least part of the action was based on a secreted product. Production of the TccC protein by *P. taiwanensis* has already been investigated for its strong insecticidal activity on several insect genera and most notably the larva of the diamond back moth *Plutella xylostella* [55]. Larvae of both *G. mellonella* and *Manduca sexta* are known to melanize and experience loss of cellular turgidity in the presence of *P. fluorescens* insecticidal toxin (Fit) [25]. Note that we did not test supernatants against nematodes or amoeba due to the nature of the tests being employed. The fast replication rate of the microbial predators would potentially have negated the ability to detect toxins that were not being renewed unless these were acutely potent.

The ability to kill insects and more effectively survive grazing suggested therefore that *Pseudomonas* encoded specific mechanisms for these phenotypes. This was directly tested by using *E. coli*-based cosmid libraries containing genomic regions of the three pathovars in the three GOT tests, to see if any of the genomic regions could enable improved *E. coli* survival. A large-scale screening was carried out testing 5800 cosmid clones in each GOT. The *Pto* library resulted in a higher number of positives (201) compared with *Pae* (101) and *Pph* (74). This does not appear to reflect over-representation of cosmids in the library because the same cosmid was not isolated repeatedly. More *Pto* cosmids were found for the nGOT, and strikingly, the iGOT analysis, whereas less *Pto* cosmids were found for aGOT. To aid the analysis, we focused on the cosmids displaying the most prominent GOT phenotypes and end sequenced 123 cosmids to map the genes found in each one. A wide range of genes with various metabolic, adhesive, virulence, cellular transport, biofilm formation, secretory, regulatory and those with an unknown function were observed with a summary of best hits from the BLAST analysis listed in Supplementary Table S2. To facilitate further investigation, we chose a sub-set of these gene systems and validated our selection by choosing only those which had been positively identified in more than one GOT assay and for more than one strain of *P. syringae*. The six more promising genes identified via this selection process were *pslA*, *fleQ*, *pilJ*, *cheW*, *rhsD* and *hlyD*. A set of six insertional knockout mutants was created in *Pto* with *cheA* and *hlyIII* mutants to replace *cheW* and *hlyD*, respectively, as surrogate functional comparators due to difficulties in chromosomal manipulation at these two loci.

The ability of *Pto::pslA* to withstand both predation by *C. elegans* and *A. polyphaga* and cause mortality in *G. mellonella* was attenuated. The *psl* gene cluster, of which *pslA* forms part of, is known to be important in the production of exopolysaccharide biosynthesis and biofilm formation [47,56]. Biofilms may act as barriers preventing ingestion of bacteria by invertebrates. A biofilm produced by *Yersinia pestis* inhibited feeding by *C. elegans* by obstructing passage to the gut [57]. Protozoan grazing of *Vibrio cholerae* was reduced in cells protected within the matrix of a biofilm whereas their planktonic counterparts were readily preyed upon [53,58]. Interestingly, the authors of this study posited that the production of the biofilm alone was not enough to dissuade predators or prevent predation but was more a combination of other secreted antiprotozoal factors. It seems possible that bacterial communities living within a biofilm, especially in lieu of quorum sensing [59,60], may be capable of modulating a concerted effort towards the increased production of virulence effectors adversely affecting potential predators. Similarly, biofilms have been shown to protect ingested bacteria against the insect immune system [61]. Following successful initial evasion, it is possible that ingested (or in the case of this study—injected) bacteria may be able to cement themselves firmly inter- and/or intracellularly and begin the production of various virulence factors.

Whereas no differences in grazing rates by *C. elegans* and *A. polyphaga* were found in *Pto::fleQ* compared to the *Pto* wildtype; the opposite was found in the *G. mellonella* toxicity assay. Hypertoxicity of the *fleQ* mutant was thought to be attributed to an overproduction of the *fleQ*-regulated negatively-charged exopolysaccharide alginate (encoded by *algD*). This suggestion was supported by the creation of an *algD* mutant, which was completely attenuated in its virulence capability in *G. mellonella*. Alginate is one of several known virulence factors produced by *P. syringae* which facilitate proliferation of infections upon colonization of plants [37]. Although there is some indication of the importance of *algD* in virulence of *P. aeruginosa* in CF patients [35] there is very little evidence of its role in invertebrate infection. Indeed, a *P. aeruginosa algD* mutant was not impaired in virulence of either plants, nematodes or mice [62]. Conversely, a *P. fluorescens* NZ17 *algU/mucA* transposon mutant expressed reduced nematode repellency compared to its wild-type [15] and AlgU, an RNA polymerase sigma factor, is necessary for the expression of mucoidy and *algD* transcription [63]. As production of alginate is known to be highly associated with biofilm formation in *P. syringae* [64] it is possible that *pslA* and *algD* work cooperatively to cause hypervirulence in *G. mellonella*.

Both nGOT and aGOT assays revealed how the pilus forming associated gene, *pilJ*, increased resistance to predation with a knockout *Pto::pilJ* mutant exhibiting lowered survivability in *C. elegans* and *A. polyphaga*. PilJ is a chemosensory protein which interacts directly with PilA—an important subunit in the overall pilin structure [65]. Pili are well known for their abilities to promote bacterial adhesion both to various biotic [66,67,68], and abiotic surfaces [69]. This adhesion is considered to be the initial step in the formation of a biofilm crucial to the survival and indeed virulence of many microorganisms in their respective hosts or environments [70]. In *P. aeruginosa* signal transduction regulating cAMP production and transcription of a large number of virulence genes followed contact of type IV pili with solid surfaces [65]. Whilst the role of pili in bacterial virulence in animal models is well characterized, in this study, interestingly, the *pilJ* mutant was not attenuated in its ability to cause mortality in *G. mellonella*. On the other hand, pathogens including a *Burkholderia pseudomallei pilA* mutant had reduced adherence to human epithelial cells and was found to be less virulent in *C. elegans* [71].

Pili linked genes are known to be highly associated with the *che* gene cluster [72] and the *pil-chp (che)* pathway controls type IV pilus production and twitching motility in *P. aeruginosa* [73]. Both *pil* and *che* gene clusters have been shown to be essential in chemotaxis in *P. aeruginosa* [74] and in turn modulate flagella expression and activity. Via methyl-accepting chemotaxis proteins CheW sends a signal to the histidine protein kinase CheA which ultimately stimulates rotation of the flagella in *P. syringae* [75]. While the primary role of flagella is for locomotion they also serve as important chemosensory organelles, adhesins and virulence factors facilitating invasion into host tissue [76,77]. Due to difficulties in creating a *cheW* mutant a closely associated *cheA* mutant was created to act as a surrogate in survival and virulence trials. The *Pto::cheA* mutant exhibited lowered survivability in *C. elegans* and *A. polyphaga* but, interestingly, no effect on *G. mellonella* mortality was observed. In a similar study, the closely associated methyltransferase CheB2, required for mobility and chemotaxis in *P. aeruginosa*, was found to be critical in virulence of both *C. elegans* killing (70% attenuation in CheB2 mutant) and murine lung infection [78]. Transposon mutants in *cheA*, *cheB* and *cheR* in two *Salmonella enterica* serovars, however, revealed that these genes were of minor importance in interactions with *C. elegans* [79]. In this same study, the authors observed reduced interactions of a *fliC* mutant attenuated in ability to produce flagellin, another key element in the construction of the overall flagella machinery, with amoebae. The conflicting observations reported in these studies illustrate a differential contribution of the chemotaxis systems in three pathogens. Potentially, as an enteric, *Salmonella* has evolved alternative mechanisms for survival in the nematode. It has also been reported that identically encoded virulence factors shared across bacterial taxa do not automatically result in pathogenicity associated at these loci [80,81].

Increased survivability of *E. coli* to *C. elegans* and *A. polyphaga* was conferred by an Rhs (Rearrangement Hotspot) factor. Survival of *Pto::rhsD* was attenuated in subsequent competition trials with both predators. Virulence in *G. mellonella* was lowered (30%) but this was not statistically significant. Whilst Rhs elements are widely distributed across bacterial genera little still is known about their overall function [41] although the structure of various ABC toxin subunits are known to contain Rhs repeats [82] indicating that many Rhs proteins in the database are in fact toxins. Expression of *rhsD* appears to contribute to virulence and we speculate that this may be due to a synergistic cooperation and combined activity of various virulence factors. This suggestion is corroborated in a recent study on the plant pathogen *Xanthomonas oryzae* where XadM, a cell surface protein in the Rhs family, was found to be important in several virulence mechanisms including attachment and biofilm formation [83]. Rhs elements have also been associated with the type VI secretion system (T6SS) and in *Dickeya dadantii*, RhsA is thought to be exported via this secretion complex. The target of this secreted protein has not been clearly elucidated but it is possibly an effector protein associated with virulence.

The final gene identified in our three-pathogen screening assay was *hlyD*. HlyD belongs to a family of membrane fusion proteins involved in the transportation of α-haemolyin toxins via a type I secretion system which is common to and crucial in virulence of many Gram-negative pathogens [84]. Interestingly, *E. coli hlyD* shares highly comparable protease secretion functions with the important phytopathogen *Dickeya dadantii* (syn. *Erwinia chrysanthemi*, *Pectobacterium chrysanthemi*) [85,86] which has been recognised as a potent experimental pathogen of the pea aphid *Acyrthosiphon pisum* [21]. Although we were not able to create a *Pto::hlyD* mutant we successfully created a mutation in a related haemolysin, *hlyIII*. Whilst survival of the mutant to *C. elegans* was initially observed after a 24-h period this was no longer the case at 72 h. Remarkably, and mirroring the results recorded for *Pto::fleQ*, we found the *hlyIII* mutant was hypervirulent towards *G. mellonella*, an unexpected result. Even though there is little evidence in the literature of any direct role which HlyIII may play in virulence in vivo, a study in *Bacillus cereus* suggests that, dependent on temperature, HlyIII forms oligomeric transmembrane pores in human erythrocytes thereby lysing them [87]. 

## 5. Conclusions

In the current study we investigated the genetic mechanisms behind survival of *P. syringae* upon challenge by two invertebrate predators and toxicity towards an insect as an alternate host. Due to the ecological niche that it occupies, the likelihood that *P. syringae* encounters y these organisms is high. Armed with this knowledge we hypothesised that *P. syringae* has evolved specialist mechanisms to cope with such stressors. Although various pseudomonads are well characterized animal pathogens, with *P. aeruginosa* being the classically referenced strain known for its arsenal of potent virulence factors [88], *P. syringae* is generally referred to as a plant pathogen [89] with little or no pathogenicity towards animals. A recent study has provided evidence of the pathogenic potential of *P. syringae* MB03 towards the nematode *C. elegans* using a range of bioassays [80]. Further transcriptional assays conducted by these authors revealed a number of candidate virulence genes (namely *algU*, *algL*, *pilA*, *fliC* and *fleN*) during the infection process [80] which were either detected, likewise, in our study and are described here or are intimately associated with them. Indeed, our GOT screening identified 358 cosmids that provided weak to strong survival or toxicity phenotypes. Notwithstanding cosmid overlap and that not all genes on the cosmids are relevant, it does illustrate that optimal bacterial survival/toxicity is polygenic and complex in nature.

To our knowledge this is the first time that a study, encompassing a host-pathogen interaction assay involving three different *P. syringae* strains in three unrelated invertebrate hosts, has been conducted. A similar study testing scale of pathogenicity of 12 different *P. syringae* isolates found close correlation between epiphytic ability and virulence towards insects [90]. Interestingly the Cit7 strain [90,91], considered nonpathogenic to plants, was the most virulent towards two well characterized hemipteran insect pests. Our results give further insight into the complex interactions occurring on the surface of plants between epiphytic and potentially entomopathogenic bacteria and plausible crop pests as well as support the possibility of using *P. syringae* as a biocontrol agent in non-host plants.

## Figures and Tables

**Table 1:** Bacterial strains and invertebrates used in this study.

Organism	Abbreviation	Relevant Characteristics
Bacterial strains		
*Pseudomonas syringae* pv. *tomato* DC3000	*Pto*	Tomato pathogen [29]
*Pseudomonas syringae* pv. *aesculi* isolate P6617	*Pae*	Isolated from horse chestnut in Glasgow, UK [30]
*Pseudomonas syringae* pv. *phaseolicola* strain 1448A	*Pph*	Bean pathogen [31]
*Escherichia coli* OP50	*E. coli*	Uracil auxotroph [32]
Invertebrates		
*Acanthamoeba polyphaga*		[26]
*Caenorhabditis elegans*		[26]
*Galleria mellonella*		[26]

**Table 2:** Summary of positive cosmid colonies isolated from each GOT screen after challenge against *Caenorhabditis elegans* and *Acanthamoeba polyphaga*, and the *Galleria mellonella* killing assay.

**Degree of Resistance (days)**						
nGOT ^1^	Very low (1)	Low (3)	Medium (5)	High (7)	Very high (9)	Resistant (11)
*Pto*	20	15	5	1	5	4
*Pph*	0	0	2	3	2	2
*Pae*	5	10	3	2	4	1
aGOT ^2^	Very low (3)	Low (5)	Medium (7)	High (9)	Very high (11)	Resistant (11+)
*Pto*	15	10	4	1	2	2
*Pph*	39	5	1	6	1	2
*Pae*	25	20	0	2	1	2
**Degree of Virulence (days)**						
iGOT ^3^	Very low (0.5)	Low (1)	Medium (2)	High (3)	Very high (4)	Virulent (4+)
*Pto*	60	20	7	13	10	7
*Pph*	0	0	3	5	2	1
*Pae*	4	3	4	8	4	1

^1,2^ The numbers of cosmids were clustered for their resistance phenotypes in the *C. elegans* (nGOT) and *A. polyphaga* (aGOT) assays based on the relative disappearance of their respective bacterial colony over time. Degrees of resistance (Very low to Resistant) have been scored on a persistence over time scale; numbers in brackets represent days, e.g., (5) is 5 days. ^3^ In the *G. mellonella* (iGOT) killing assay, all cosmids were clustered on their toxicity phenotype. The degree of toxicity (Very low to Virulent) was classified on the death phenotype caused by the cosmid clone within *G. mellonella* over time; days in brackets, e.g., (2) is 2 days. The toxicity of the cosmid colonies was scored based on time to death: still alive (scored 0.5); 4 days (scored 1); 3 days (scored 2); 2 days (scored 3); 1 day (scored 4); after 12 h (scored 4+). *Pseudomonas syringae* pv. *tomato* (*Pto*), *phaseolicola* (*Pph*) and *aesculi* (*Pae*).

**Table 3:** List of mutants created following selection of target genes by Rapid Virulence Annotation (RVA) analysis. .

Mutant	Gene	Function	GOT
*Pto*::*algD*	*algD* (PSPTO_1243)	Alginate biosynthesis	Control mutant for GOT assays
*Pto*::*fleQ*	*fleQ* (PSPTO_1954)	Transcriptional regulator, flagella regulation	nGOT/iGOT
*Pto*::*pslA*	*pslA* (PSPTO_3529)	Exopolysaccharide biosynthesis and biofilm formation	nGOT/iGOT
*Pto*::*pilJ*	*pilJ* (PSPTO_5031)	Type IV pilus biogenesis	nGOT/aGOT
*Pto*::*cheA*	*cheA* (PSPTO_0913)	Chemotaxis	nGOT/aGOT (for *cheW*)
*Pto*::*rhsD*	*rhsD* (PSPTO_0373)	Rearrangement hotspot family protein	nGOT/iGOT
*Pto*::*hlyIII*	*hlyIII* (PSPTO_5077)	Haemolysin	aGOT/nGOT (for *hlyD*)

## Supplementary Materials

The following are available online at http://www.mdpi.com/2076-2607/6/2/32/s1, Table S1: List of primers used in this study, Table S2: Summary of best hits and identified gene regions from the RVA using *Pto* as a reference.
