# Supplementary material for: Coping with Environmental Eukaryotes; Identification of Pseudomonas syringae Genes during the Interaction with Alternative Hosts or Predators
Source: Microorganisms. 2018 Apr 21;6(2):32. doi: 10.3390/microorganisms6020032 (PMC6027264; doi:10.3390/microorganisms6020032)
Supplement: Supplementary file 1 [file microorganisms-06-00032-s001.pdf]

Supplementary Table S1. List of primers used in this study

Primer name	Primer Sequence	Gene Target
AlgDProFor	GAA TTG GCT TGA GGC TCG CAA CTG AC	*algD*
AlgDProREV	GCA GCA ACA CCT CGC AAG TGC GAA TT	*algD*
AlgDextFor	CAG GCT GCA AGC TCA TTG T	*algD*
AlgDextRev	AAT CGT CAG ACA GGA GTA GT	*algD*
HlyDKOfor	GAC TGG CAC CAT TGT TGA TG	*hlyD*
HlyDKOrev	ATC TTT TGA TCG GCT TGC TG	*hlyD*
hlyIIIKOfor	TGC GAT TTG GTT ATT GGT GA	*hlyIII*
hlyIIIKOrev	TTC GGA ACG GGG TTT TAT CT	*hlyIII*
T3	AAT TAA CCC TCA CTA AAG GG	piJ3200 MCS*
T7	TAA TAC GAC TCA CTA TAG GG	piJ3200 MCS
PilJKO_For	CCG TGA CGG CTT CTG TTA CCG	*pilJ*
PilJKO_Rev	CTC ACC CAA GCG TTT GAT GCG	*pilJ*
pLAFR3Forint	GAG TTA GCT CAC TCA TTA GGC	pLAFR3 MCS
pLAFR3IntRev	TGT AAA ACG ACG GCC AGT GCC	pLAFR3 MCS
Primer0372_For2	GCG GCA ATG AGG ACG TCC GAC TGA GAT GGT CAG CCC GAT CC	PSPTO_0373
Primer0372_For2	GCG GCA ATG AGG ACG TCC GAC TGA GAT GGT CAG CCC GAT CC	PSPTO_0373
PSPTO0373for	TGC GTT ACC ACT ACG ACA GC	PSPTO_0373
PSPTO0373rev	ACT CAA AGC CCA GCT GAT TG	PSPTO_0373
M13 rev (-29)	CAG GAA ACA GCT ATG ACC	Universal Primer
M13 uni (-21)	TGT AAA ACG ACG GCC AGT	Universal Primer

*MCS – Multiple Cloning Site

Supplementary Table S2. Summary of best hits and identified gene regions from the RVA using *Pto* as a reference.

				Found in other RVA screenings	
**Gene ID**	**Gene Description**	**Additional Information**	**GOT**	***Pph***	***Pae***
**PSPTO 3616**	Hypothetical Protein (Conserved Hypothetical Protein)	putative peptidase	nGOT		
**PSPTO 3617**	transcriptional regulator, MarR family		nGOT		
**PSPTO 3618**	membrane protein, putative	fusaric acid resistance protein region	nGOT		
**PSPTO 3619**	membrane protein, putative	Unknown function	nGOT		
**PSPTO 3620**	HlyD family secretion protein	HlyD, a member of the membrane fusion protein family	nGOT		
**PSPTO 3621**	outer membrane efflux protein	NodT family outer membrane efflux lipoprotein	nGOT		

**PSPTO 3622**	Hypothetical Protein	Unknown	nGOT		
**PSPTO 3623**	Hypothetical Protein	Unknown	nGOT		
**PSPTO 3624**	iron transport protein, putative	ion transport protein, putative	nGOT		
**PSPTO 3625**	lipoprotein, putative	sulfate ABC transporter, periplasmic sulfate-binding protein	nGOT		
**PSPTO 0373**	RhsD	Rhs family protein	nGOT/aGOT	+	
**PSPTO 5414**	lipoprotein, putative	Unknown	nGOT		
**PSPTO 5415**	Rhs element Vgr protein		nGOT		
**PSPTO 5416**	serine/threonine protein kinase, putative		nGOT		
**PSPTO 5417**	serine/threonine phosphoprotein phosphatase		nGOT		
**PSPTO 5418**	conserved hypothetical protein	type VI secretion protein VasK	nGOT	+	+
**PSPTO 5419**	conserved hypothetical protein	putative type VI secretion protein VasF	nGOT	+	+
**PSPTO 5420**	conserved hypothetical protein	putative type VI secretion protein VasE	nGOT	+	+
**PSPTO 5421**	lipoprotein, putative	putative type VI secretion protein VasD	nGOT	+	+
**PSPTO 5422**	FHA domain protein	type VI secretion system FHA domain	nGOT	+	+
**PSPTO 5424**	sigma-54 dependent transcriptional regulator	Fis family transcriptional regulator	nGOT	+	+
**PSPTO 5425**	ClpB protein, putative	type VI secretion system ClpB chaperone	nGOT	+	+
**PSPTO 5426**	conserved hypothetical protein	putative type VI secretion protein VasG	nGOT	+	+
**PSPTO 5427**	conserved hypothetical protein	type VI secretion protein, family VasA	nGOT	+	+
**PSPTO 5428**	ISP5 Transposase		nGOT		
**PSPTO 5429**	Hypothetical Protein	type VI secretion system protein vasH	nGOT	+	+
**PSPTO 5430**	Hypothetical Protein	PAAR	nGOT	+	+
**PSPTO 5431**	Hypothetical Protein	type VI secretion system lysozyme-related	nGOT	+	+
**PSPTO 5432**	Hypothetical Protein	Putative type VI secretion protein VasR	nGOT	+	+
**PSPTO 5433**	Hypothetical Protein	Putative type VI secretion protein VasQ	nGOT	+	+
**PSPTO 5434**	Conserved hypothetical protein	Putative type VI secretion protein VasJ	nGOT	+	+
**PSPTO 5435**	Secreted protein Hcp	secreted protein Hcp	nGOT	+	+
**PSPTO 5436**	Rhs element Vgr protein	Rhs element Vgr protein	nGOT	+	+
**PSPTO 5437**	Hypothetical Protein	Hypothetical Protein	nGOT	+	+
**PSPTO 5438**	Rhs family protein	Rhs family protein	nGOT	+	+
**pvsA**	pyoverdine chromophore precursor synthetase		nGOT		

**daT**	2,4-diaminobutyrate 4-transaminase		nGOT		
**PSPTO 2137**	MbtH-like protein		nGOT		
**PSPTO 2138**	ABC transporter, periplasmic substrate-binding protein, putative		nGOT		
**PSPTO 2139**	cation ABC transporter, permease protein		nGOT		
**PSPTO 2140**	cation ABC transporter, ATP-binding protein		nGOT		
**PSPTO 2141**	cation ABC transporter, periplasmic cation-binding protein		nGOT		
**PSPTO 2142**	conserved hypothetical protein		nGOT		
**PSPTO 2143**	conserved hypothetical protein		nGOT		
**PSPTO 2144**	conserved hypothetical protein		nGOT		
**PSPTO 2145**	iron-regulated membrane protein, putative		nGOT		
**PSPTO 2146**	pyoverdine biosynthesis regulatory gene, putative		nGOT		
**PSPTO 2147**	pyoverdine sidechain peptide synthetase I, epsilon-Lys module		nGOT		
**PSPTO 2148**	pyoverdine sidechain peptide synthetase II, D-Asp-L-Thr component		nGOT		
**PSPTO 2149**	pyoverdine sidechain peptide synthetase III, L-Thr-L-Ser component		nGOT		
**PSPTO 2150**	pyoverdine sidechain peptide synthetase IV, D-Asp-L-Ser component		nGOT		
**PSPTO 2151**	TonB-dependent siderophore receptor, putative		nGOT		
**PSPTO 2152**	TonB-dependent siderophore receptor, putative		nGOT		
**PSPTO_4339**	fumarate hydratase, class I, putative		nGOT		
**PSPTO_4340**	insecticidal toxin protein, putative	TccC3	nGOT	+	+
**PSPTO_4341**	insecticidal toxin protein, putative		nGOT	+	+
**PSPTO_4342**	insecticidal toxin protein, putative	TccB2	nGOT	+	+
**PSPTO_4343**	insecticidal toxin protein, putative	virulence B protein (similar to TccA *Photorhabdus*)	nGOT		
**PSPTO_4344**	insecticidal toxin protein, putative	TccC3	nGOT	+	+
**PYK**	pyk pyruvate kinase		nGOT		
**PSTP_1961**	flagellum-specific ATP synthase FliI		nGOT	+	+

**PSPTO_1962**	flagellar protein FliJ, putative	nGOT	+	+
**PSPTO_1963**	STAS domain protein	nGOT	+	+
**PSPTO_1964**	response regulator	nGOT	+	+
**PSPTO_1965**	Hypothetical Proteint domain protein	nGOT	+	+
**Flik**	flagellar hook-length control protein FliK	nGOT	+	+
**PSPTO_1967**	hypothetical protein	nGOT	+	+
**PSPTO_1968**	flagellar protein FliL, putative	nGOT	+	+
**FliM**	flagellar motor switch protein FliM	nGOT/iGOT	+	+
**FliN**	flagellar motor switch protein FliN	nGOT/iGOT	+	+
**FliO**	flagellar protein FliO	nGOT/iGOT	+	+
**FliP**	flagellar protein FliP	nGOT/iGOT	+	+
**FliQ**	flagellar biosynthetic protein FliQ	nGOT/iGOT	+	+
**FliR**	fliR flagellar biosynthetic protein FliR	nGOT/iGOT	+	+
**FlhB**	flhB flagellar biosynthetic protein FlhB	nGOT/iGOT	+	+
**FlhA**	flhA flagellar biosynthetic protein FlhA	nGOT/iGOT	+	+
**FlhF**	flhF flagellar biosynthetic protein FlhF	nGOT/iGOT	+	+
**FleN**	fleN flagellar synthesis regulator FleN	nGOT/iGOT	+	+
**FliA**	fliA motility sigma factor FliA	nGOT/iGOT	+	+
**PSPTO_0809**	hydroxymethylbutenyl pyrophosphate reductase	nGOT/aGOT	+	
**PSPTO_0810**	type IV pilus biogenesis protein	nGOT/aGOT	+	
**PSPTO_0811**	pillin, putative	nGOT/aGOT	+	
**PSPTO_0812**	hypothetical protein	nGOT/aGOT	+	
**PSPTO_0813**	hypothetical protein	nGOT/aGOT	+	
**PSPTO_0814**	conserved hypothetical protein	nGOT/aGOT	+	
**PSPTO_0815**	type IV pilus-associated protein, putative	nGOT/aGOT	+	
**PSPTO_0816**	type IV pilus biogenesis protein	nGOT/aGOT	+	
**PSPTO_0817**	oxidoreductase, FAD-binding	nGOT/aGOT	+	
**PSPTO_0818**	transcriptional regulator, MarR family	nGOT/aGOT	+	
**PSPTO_0819**	conserved hypothetical protein	nGOT/aGOT	+	
**PSPTO_0820**	AcrB/AcrD/AcrF family protein	nGOT/aGOT	+	

**PSPTO_0821**	efflux transporter, RND family, MFP subunit	nGOT/aGOT	+
**PSPTO_0822**	transcriptional regulator, TetR family	nGOT/aGOT	+
**PSPTO_0823**	4 fimbriae expression regulatory protein pilR	nGOT/aGOT	+
**PSPTO_0824**	sensor protein PilS	nGOT/aGOT	+
**PSPTO_0825**	conserved hypothetical protein	nGOT/aGOT	+
**PSPTO_0826**	competence lipoprotein ComL, putative	nGOT/aGOT	+
**PSPTO_1170**	conserved hypothetical protein	nGOT	
**pta**	phosphate acetyltransferase	nGOT	
**PSPTO_1171**	peptidyl-prolyl cis-trans isomerase, FKMBP-type	nGOT	
**PSPTO_1172**	Glutathione peroxidase family protein	nGOT	
**PSPTO_1173**	oxidoreductase, FAD/FMN-binding	nGOT	
**PSPTO1174**	glycosyl transferase, group 1 family protein	nGOT	
**PSPTO1175**	Membrane protein, putative	nGOT	
**PSPTO_1176**	conserved domain protein	nGOT	
**cysZ**	cysZ protein	nGOT	
**trxB**	Thioredoxin reductase	nGOT	
**hopJ1**	type III effector HopJ1	nGOT	+
**folB-2**	folB-2 dihydroneopterin aldolase	nGOT	
**folE-1 GTP**	folE-1 GTP cyclohydrolase I	nGOT	
**PSPTO_1183**	oxidoreductase, short chain dehydrogenase/reductase family	nGOT	
**PSPTO_1186**	Flavodoxin	nGOT	
**PSPTO_2099**	helicase/SNF2 family domain protein	nGOT	
**PSPTO_2098**	isochorismatase family protein	nGOT	
**PSPTO_2097**	oxidoreductase, short chain dehydrogenase/reductase family	nGOT	
**PSPTO_2096**	hypothetical protein	nGOT	
**PSPTO_2095**	conserved domain protein	nGOT	
**PSPTO_2093**	lysozyme, putative	nGOT	
**PSPTO_2092**	tail fiber domain protein	nGOT	
**PSPTO_2091**	conserved domain protein	nGOT	
**PSPTO_2090**	hypothetical protein	nGOT	

**PSPTO_2089**	host specificity protein J, internal deletion		nGOT		
**PSPTO_2088**	conserved hypothetical protein		nGOT		
**PSPTO_2083 to PSPTO_2087**	hypothetical protein		nGOT		
**PSPTO_2082**	tail length tape meausure protein, internal deletion		nGOT		
**PSPTO 3565**	gluconate permease		nGOT/iGOT		
**PSPTO_3564**	Gluconokinase		nGOT/iGOT		
**PSPTO_3563**	gluconate utilization system GNT-I transcriptionl repressor		nGOT/iGOT		
**PSPTO_3561**	WD40 Superfamily Hypothetical Protein		nGOT/iGOT		
**PSPTO_3560**	GDA1/CD39 family protein		nGOT/iGOT		
**PSTPO 3555 to PSPTO3559**	*glc* genes biosynthesis cluster	Oxalate product fromGlycolate biosyntehesis toxic for *C.elegans*	nGOT/iGOT	+	+
**PSTPO 3350 to PSPTO3555**	Melanin biosynthesis cluster	Gene to product Homogentisate Melain precursor	nGOT/iGOT		
**PSPTO 3548**	Hypothetical Protein (Conserved Hypothetical Prot)	B lactamase superfamily	nGOT/iGOT		
**PSPTO 3547**	Trasctipt Regulator LysR family		nGOT/iGOT		
**PSPTO_3544**	Hypothetical Protein (Conserved HypotheticalProt)	Probabily thirosine kinase fam	nGOT/iGOT		
**PSPTO_3543**	Hypothetical Protein (Conserved HypotheticalProt)	Unknown	nGOT/iGOT		
**PSPTO_3542**	Hypothetical Protein (Conserved HypotheticalProt)	Unknown	nGOT/iGOT		
**PSPTO_3539**	*mivN*	*Virulence* gene in *Salmonella* and *E. coli*	nGOT/iGOT		
**PSPTO_3538**	CHypothetical Protein (Conserved HypotheticalProt)	bacterial transferase, hexapeptide repeat protein	nGOT/iGOT		
**PSPTO_3537**	*pslK*	Exopolysaccharide biosynthesis and biofilm formation in *P. aeruginosa*	nGOT/iGOT	+	+
**PSPTO_3536**	*pslJ*	Exopolysaccharide biosynthesis and biofilm formation in *P. aeruginosa*	nGOT/iGOT	+	+
**PSPTO_3535**	*pslH*	Exopolysaccharide biosynthesis and biofilm formation in *P. aeruginosa*	nGOT/iGOT	+	+
**PSPTO_3534**	*pslG*	Exopolysaccharide biosynthesis and biofilm formation in *P. aeruginosa*	nGOT/iGOT	+	+
**PSPTO_3533**	*pslF*	Exopolysaccharide biosynthesis and biofilm formation in *P. aeruginosa*	nGOT/iGOT	+	+
**PSPTO_3532**	*pslE*	Exopolysaccharide biosynthesis and biofilm formation in *P. aeruginosa*	nGOT/iGOT	+	+

**PSPTO_3531**	*pslC*	Exopolysaccharide biosynthesis and biofilm formation in *P. aeruginosa*	nGOT/iGOT	+	+
**PSPTO_3530**	*pslB*	Exopolysaccharide biosynthesis and biofilm formation in *P. aeruginosa*	nGOT/iGOT	+	+
**PSPTO 3529**	*pslA*	Exopolysaccharide biosynthesis and biofilm formation in *P. aeruginosa*	nGOT/iGOT	+	+
**PSPTO_1563**	*pcM*	protein-L-isoaspartate O-methyltransferase	nGOT/iGOT		
**PSPTO_1562**	*surE*	stationary-phase survival protein SurE	nGOT/iGOT		
**PSPTO_1561**	Hypothetical Protein	tRNA pseudouridine synthase D	nGOT/iGOT	+	+
**PSPTO_1560**	*ispF*	2C-methyl-D-erythritol 2,4-cyclodiphosphate	nGOT/iGOT		
**PSPTO_1559**	Hypothetical Protein	Esterase	nGOT/iGOT		
**PSPTO_1558**	*adc*	OH dehyrogenase	nGOT/iGOT		
**PSPTO_1557**	transcriptional regulator, LysR family		nGOT/iGOT		
**PSPTO_1556**	*ispD*	4-diphosphocytidyl-2C-methyl-D-erythritol synthase	nGOT/iGOT		
**PSPTO_1555**	Hypothetical Protein	cell division protein FtsB	nGOT/iGOT		
**PSPTO_1554**	*eno*	enolase	nGOT/iGOT		
**PSPTO_1553**	*kdsa*	2-dehydro-3-deoxyphosphooctonate aldolase	nGOT/iGOT		
**PSPTO_1552**	*pyrG*	CTP synthase	nGOT/iGOT		
**PSPTO_1551**	PSPTO_1551	cell cycle protein mesJ, putative	nGOT/iGOT		
**PSPTO_1550**	*accA*	acetyl-CoA carboxylase, carboxyl transferase, alpha subunit	nGOT/iGOT		
**PSPTO_1549**	*dnaE*	DNA polymerase III, alpha subunit	nGOT/iGOT		
**PSPTO_1548**	*rnhB*	ribonuclease HII	nGOT/iGOT		
**PSPTO_1547**	*lpxB*	lipid A disaccharide synthase	nGOT/iGOT		
**PSPTO_1546**	*lpxA*	acyl-[acyl-carrier-protein]--UDP-N-acetylglucosamine O-acyltransferase	nGOT/iGOT		
**PSPTO_1545**	*fabZ*	beta-hydroxyacyl-(acyl-carrier-protein) dehydratase FabZ	nGOT/iGOT		
**PSPTO_1544**	*ipxD*	UDP-3-O-[3-hydroxymyristoyl] glucosamine N-acyltransferase	nGOT/iGOT		
**PSPTO_1543**	PSPTO_1543	outer membrane protein OmpH, putative	nGOT/iGOT	+	+
**PSPTO_1542**	PSPTO_1542	outer membrane protein	nGOT/iGOT	+	+
**PSPTO_1541**	PSPTO_1541	membrane-associated zinc metalloprotease, putative	nGOT/iGOT		
**PSPTO_1540**	*dxr*	1-deoxy-D-xylulose 5-phosphate reductoisomerase	nGOT/iGOT		

**PSPTO_1539**	*cds*	phosphatidate cytidylyltransferase	nGOT/iGOT		
**PSPTO_1538**	*upps*	undecaprenyl diphosphate synthase	nGOT/iGOT		
**PSPTO_1537**	*frr*	ribosome recycling factor	nGOT/iGOT		
**PSPTO_1536**	*pyrH*	uridylate kinase	nGOT/iGOT		
**PSPTO_1535**	*tsf*	translation elongation factor Ts	nGOT/iGOT		
**PSPTO_1534**	*rpsB*	ribosomal protein S2	nGOT/iGOT		
**PSPTO 3611**	ISPsy*5*		nGOT/aGOT		
**PSPTO 3612**	ISPsy*5*		nGOT/aGOT		
**PSPTO 3613**	ISPsy*5*		nGOT/aGOT		
**PSPTO 3615**	site-specific recombinase, phage integrase family		nGOT/aGOT		
**PSPTO 3616**	Hypothetical Protein	putative peptidase	nGOT/aGOT		
**PSPTO 3617**	transcriptional regulator, MarR family		nGOT/aGOT		
**PSPTO 3619**	Membrane protein, putative	fusaric acid resistance protein region	nGOT/aGOT		
**PSPTO 3620**	HlyD family secretion protein		nGOT/aGOT		
**PSPTO 3621**	outer membrane efflux protein	NodT family outer membrane efflux lipoprotei	nGOT/aGOT		
**PSPTO 3622**	Hypothetical Protein	Unknown	nGOT/aGOT		
**PSPTO 3623**	Hypothetical Protein	Unknown	nGOT/aGOT		
**PSPTO 3624**	ion transport protein, putative	ion transport protein, putative	nGOT/aGOT		
**PSPTO 3625**	lipoprotein, putative	sulfate ABC transporter, periplasmic sulfate-binding protein	nGOT/aGOT		
**PSPTO_5191**	AcrB/AcrD/AcrF family protein		nGOT/aGOT		
**PSPTO_5192**	efflux transporter, RND family, MFP subunit		nGOT/aGOT		
**PSPTO_5194**	conserved domain protein	Rhs family protein [*Yersinia intermedia* ATCC 29909	nGOT/aGOT	+	+
**PSPTO_5195**	ABC transporter, periplasmic substrate-binding protein		nGOT/aGOT		
**PSPTO_5196**	ABC transporter, permease protein		nGOT/aGOT		
**PSPTO_5197**	ABC transporter, ATP-binding protein		nGOT/aGOT		
**PSPTO_5198**	dioxygenase, TauD/TfdA family		nGOT/aGOT		
**PSPTO_5199**	Hypothetical Protein	Hypothetical Protein	nGOT/aGOT		
**PSPTO_5200**	autotransporter, putative		nGOT/aGOT		
**PSPTO_5202**	Hypothetical Protein	enhanced entry protein EnhC [*Legionella pneumophila* str. Paris] 30%	nGOT/aGOT	+	+

**PSPTO_5203**	Hypothetical Protein	EF hand domain-containing protein	nGOT/aGOT	+	+
**PSPTO_5204**	EF hand domain protein	EF hand domain protein	nGOT/aGOT	+	+
**PSPTO_5205**	Hypothetical Protein	EF hand domain protein	nGOT/aGOT	+	+
**PSPTO_5206**	EF hand domain protein	EF hand domain protein	nGOT/aGOT	+	+
**PSPTO_5207**	Hypothetical Protein	Secreted UNKNOW FUNCTION	nGOT/aGOT	+	+
**PSPTO_5208**	Hypothetical Protein	EF hand domain protein	nGOT/aGOT	+	+
**PSPTO_5209**	Hypothetical Protein	EF hand domain protein	nGOT/aGOT	+	+
**PSPTO_5210**	EF hand domain protein	EF hand domain protein	nGOT/aGOT	+	+
**PSPTO_5211**	Hypothetical Protein	putative secreted protein Unknown FUNCTION	nGOT/aGOT	+	+
**PSPTO_5212**	ISPsy*5*, transposase		nGOT/aGOT		
**PSPTO_5213**	ISPsy*5*, Orf1		nGOT/aGOT		
**PSPTO_5214**	ISPsy*5*, Orf1		nGOT/aGOT		
**PSPTO_5215**	ISPsy*5*, transposase		nGOT/aGOT		
**PSPTO_1493**	methyl-accepting chemotaxis protein	methyl-accepting chemotaxis protein	nGOT/aGOT	+	
**PSPTO_1494**	CheW domain protein	CheW domain protein	nGOT/aGOT	+	
**PSPTO_1495**	chemotaxis protein methyltransferase CheR, putative	chemotaxis protein methyltransferase CheR, putative	nGOT/aGOT	+	
**PSPTO_1496**	CheW domain protein	CheW domain protein	nGOT/aGOT	+	
**PSPTO_1497**	sensor histidine kinase/response regulator	sensor histidine kinase/response regulator	nGOT/aGOT	+	
**PSPTO_1498**	protein-glutamate methylesterase CheB	protein-glutamate methylesterase CheB	nGOT/aGOT	+	
